# Implementing Interventions with Varying Marginal Cost-Effectiveness: An Application in Precision Medicine

**DOI:** 10.1177/0272989X20954391

**Published:** 2020-10-21

**Authors:** Stuart J. Wright, Mike Paulden, Katherine Payne

**Affiliations:** Manchester Centre for Health Economics, Division of Population Health, Health Services Research & Primary Care, University of Manchester, Manchester, Greater Manchester, UK; School of Public Health, University of Alberta, Edmonton, AB, Canada; Manchester Centre for Health Economics, Division of Population Health, Health Services Research & Primary Care, University of Manchester, Manchester, Greater Manchester, UK

**Keywords:** breast cancer, economic evaluation, implementation, precision medicine

## Abstract

**Purpose**. A range of barriers may constrain the effective implementation of strategies to deliver precision medicine. If the marginal costs and consequences of precision medicine vary at different levels of implementation, then such variation will have an impact on relative cost-effectiveness. This study aimed to illustrate the importance and quantify the impact of varying marginal costs and benefits on the value of implementation for a case study in precision medicine. **Methods**. An existing method to calculate the value of implementation was adapted to allow marginal costs and consequences of introducing precision medicine into practice to vary across differing levels of implementation. This illustrative analysis used a case study based on a published decision-analytic model-based cost-effectiveness analysis of a 70-gene recurrence score (MammaPrint) for breast cancer. The impact of allowing for varying costs and benefits for the value of the precision medicine and of implementation strategies was illustrated graphically and numerically in both static and dynamic forms. **Results**. The increasing returns to scale exhibited by introducing this specific example of precision medicine mean that a minimum level of implementation (51%) is required for using the 70-gene recurrence score to be cost-effective at a defined threshold of €20,000 per quality-adjusted life year. The observed variation in net monetary benefit implies that the value of implementation strategies was dependent on the initial and ending levels of implementation in addition to the magnitude of the increase in patients receiving the 70-gene recurrence score. In dynamic models, incremental losses caused by low implementation accrue over time unless implementation is improved. **Conclusions**. Poor implementation of approaches to deliver precision medicine, identified to be cost-effective using decision-analytic model-based cost-effectiveness analysis, can have a significant economic impact on health systems. Developing and evaluating the economic impact of strategies to improve the implementation of precision medicine will potentially realize the more cost-effective use of health care budgets.

Precision (or stratified or personalized) medicine^1^ has been defined as “an emerging approach for disease treatment and prevention that takes into account individual variability in genes, environment, and lifestyle for each person.”^2^ In practice, precision medicine is underpinned by the premise that it is feasible to identify known heterogeneity within a disease or population and use this information to guide management strategies to improve health and well-being. Precision medicine, therefore, requires a mechanism (“test” or “tool”) that, in theory and in practice, provides information in addition to the currently available strategies used to select interventions, which can be, for example, “prognostic markers, predictors of toxicities and any parameter such as environmental and lifestyle factors.”^3^ These tools can be used to identify information about a patient and their disease to predict potential improved or reduced response to treatments, such as *KRAS* gene mutation testing to target cetuximab,^3^ or higher risk of side effects, such as *CYP2C19* testing to guide the dose of warfarin to reduce major bleeding complications.^4^ Precision medicine, in general, and the use of tests to inform the prescribing of medicines (test-treat interventions), specifically, have been suggested to have economic benefits by targeting interventions only to those patients who will accrue benefits and/or are less likely to experience severe adverse drug reactions.^5^

Cost-effectiveness analysis can provide evidence about whether new health care interventions, such as test-treat interventions, represent a good investment by generating more health for patients receiving precision medicine compared with the health lost by those from whom funding is denied or removed.^6^ Decision-analytic model-based cost-effectiveness analysis (hereafter CEA) is the cornerstone of the evidence base in health technology assessment (HTA) reports produced as part of national decision making processes by bodies such as the National Institute for Health and Care Excellence (NICE)^7^ and the Canadian Agency for Drugs and Technologies in Health (CADTH).^8^ A common, but not usually explicitly stated, assumption underpinning existing CEA is that marginal costs and benefits are constant.^9,10^ This assumption of constant marginal changes implies that the mean incremental costs and incremental benefits of providing 1 more patient with the new intervention, such as a test-treat intervention, when compared with each comparator are the same regardless of the number of patients who are treated. The expected mean incremental cost and incremental benefit of the intervention for each patient is, therefore, assumed to represent the relative estimated cost-effectiveness of providing the test-treat intervention to all patients within a relevant population.

Existing applications of CEA also assume that the test-treat interventions under evaluation are divisible.^11^ Being divisible implies that it is possible to allocate precision medicine to a defined proportion of the relevant population. In practice, HTA agencies tend to evaluate new test-treat interventions with the assumption that they are not divisible to promote equity. Actioning this assumption results in 2 distinct scenarios in which either all (or none) of the relevant population will receive the specified test-treat intervention.^12^ In some circumstances, the definition of the relevant population may be a clinically prespecified subgroup of the population, for example, a group of patients with a specific genetic mutation or a known level of disease severity. It has been argued that due to these assumptions, the cost-effectiveness estimates produced by conventional economic evaluations represent an estimate of the long-run cost-effectiveness of precision medicine.^13,14^ However, in the short run, these assumptions may not be realistic and the cost-effectiveness of precision medicine may differ as a result.

It is possible, but improbable, that the entire relevant population will have access to a test-treat intervention immediately following a positive HTA recommendation. A scenario of delayed, imperfect uptake is relevant to any intervention, but precision medicine provides an exemplar of some specific challenges. A number of barriers (or capacity constraints) have been identified that may hinder fully comprehensive and inclusive access to the introduction of precision medicine: lack of sufficient laboratories to perform testing, logistical issues with coordinating testing and treatment, and sufficient numbers of trained laboratory staff, allied health care professionals, and clinicians.^15–18^ Concerns about the capacity of the National Health Service in England (NHS England) to provide the required *EGFR* mutation testing were raised during a NICE Technology Appraisal of gefitinib for non–small cell lung cancer.^19^ Despite assurances at the time of the appraisal, subsequent evidence in 2014 suggested that only around 50% of eligible patients were receiving *EGFR* testing.^20^ Issues such as geographical differences in the type of test being offered and long turnaround times for test results resulting in delayed treatment were a subsequent feature of NICE technology appraisals for erlotinib and afatanib.^21,22^ Such capacity constraints potentially impede the comprehensive and inclusive evidence-based introduction (hereafter termed *implementation*) of precision medicine with its required combination of “test” and subsequent “treatment.”

Methods are available to quantify the expected value of the improved implementation of health care interventions using specified strategies, such as investing in the required equipment or staff knowledge.^23,24^ Such approaches are henceforth referred to as “implementation strategies.” The evaluation of implementation strategies by estimating the incremental cost-effectiveness ratio was originally proposed by Sculpher^25^ in 2000 and further developed by Mason et al.^26^ in 2001. However, a move in 2007 to propose the use of the net benefit framework by Fenwick et al.,^27^ formulated by Walker et al.,^28^ allowed the calculation of the economic benefit of increasing implementation rather than a sole focus on quantifying the relative cost-effectiveness of specific implementation strategies.

Underpinning these methods is the assumption that improving implementation results in a net health benefit from the intervention under evaluation. The gain in net health benefit of improvement in implementation is then compared with the cost of the implementation strategy (activities of the health system to improve implementation) to quantify whether it is an appropriate use of health care system resources by estimating the value of implementation for a defined patient population and health care budget (VOImp). Two existing (“traditional”) value of implementation frameworks (VOImp) are available that estimate static (VOImp [static]) and dynamic (VOImp [dynamic]) values.^28^ The static approach can be used to calculate the value of using a one-time only implementation strategy to improve the implementation of a precision medicine that is cost-effective. The static approach assumes that all the impact occurs in the first year. The dynamic approach allows the costs and effects of the implementation strategy to improve implementation of a specific intervention across multiple time periods. In addition, methods exist to inform decisions as to whether to invest in implementation or additional research when faced with uncertainty in the parameters used to generate estimates of cost-effectiveness.^29–31^

To date, there have been few actual applications of VOImp, and each has focused on estimating a “static” value,^23,24,32^ and none has focused on the impact on precision medicine. Whyte et al.^23^ estimated the value of using a 2-hour training workshop for clinicians to increase the implementation of NICE guidelines for natriuretic peptide testing. Mewes et al.^24^ calculated the incremental net benefit of implementation activities to increase the use of novel oral anticoagulants to prevent strokes. This study extended the VOImp framework to understand the value of implementation in different subgroups of the patient population. Faria et al.^32^ estimated the VOImp of strategies to improve adherence to exercise guidelines for cancer survivors.

While VOImp methods can aid in decision making regarding the value of implementation strategies, to date, no VOImp study has accounted for the potential presence of varying marginal costs and benefits that are likely to be a feature of many examples of precision medicine that rely on test-treat strategies. If the marginal costs and benefits of precision medicine vary, then the cost-effectiveness and value of precision medicine will vary depending on the degree of implementation. This study aimed to illustrate the importance and quantify the impact of varying marginal costs and benefits on the value of implementation for a case study in precision medicine. Existing methods to calculate VOImp, using the static and dynamic forms, are applied to illustrate the potential impact of accounting for varying marginal costs and benefits on the relative cost-effectiveness and value of a specific exemplar of precision medicine to show how it may be applied to precision medicine more generally.

## Methods

This study uses an adaptation of decision-analytic model-based cost-effectiveness analysis to understand the impact of the value of implementation on precision medicine. This section first describes the 2 existing (“traditional”) value of implementation frameworks (VOImp), in both static (VOImp [static]) and dynamic (VOImp [dynamic]) forms, that each assume constant marginal costs and benefits.^28^ The second part of this section describes how the existing VOImp (static) and VOImp (dynamic) can be modified to allow for varying marginal costs and benefits to take account of the potential impact of capacity constraints on the relative cost-effectiveness of precision medicine. The third section describes how the 2 modified VOImp frameworks (static and dynamic) were applied to a case study in precision medicine to demonstrate the potential impact of allowing for changes in the marginal costs and benefits when assessing the relative cost-effectiveness of an example test-treat strategy.

### Traditional Value of Implementation with Constant Marginal Costs and Benefits: Static Form

The value of implementation framework in the static form (VOImp [static]) values the improved net benefit that arises from treating an expanding number of patients following an implementation strategy in a single time period. The first step in estimating the VOImp (static) involves calculating the net monetary benefit (NMB) of the intervention (see equation (1)):

(1) NMB=k.ΔH−ΔC.

Equation (1) shows how a monetary value per additional patient treated is calculated by multiplying the incremental health gained by treating patients with the new interventions compared to a comparator (ΔH) by a defined a threshold value (k) specified for accruing those gains. The threshold represents a cutoff value for the relative cost-effectiveness of the intervention compared with current practice, for example. £20,000 per quality-adjusted life year (QALY) gained.^33^ Any measure of health can be used, but most published value of implementation studies have used the QALY as the measure of health. The incremental cost of treating patients with the intervention rather than the comparator (ΔC) is then subtracted from the health gain represented as a monetary value. Note that in the VOImp (static) framework, the calculated NMB will be the same for each additional patient, regardless of the level of implementation.

The current value of implementation represents the value of the intervention to society in terms of the total incremental net benefit it provides given the proportion of patients receiving the intervention at the current time. This (see equation (2)) is calculated by multiplying the value of NMB by the total number of patients (*n*) in the relevant population and the proportion of those patients receiving the intervention at the current implementation level (*p*):

(2) n.p.NMB.

The monetary value of increasing to full implementation (see equation (3)) is then calculated by subtracting the number of patient population currently receiving the intervention (1 –*p*) from the NMB multiplied by the total patient population:

(3) n.(1−p).NMB.

The resulting value represents the potential VOIMp (static) associated with increasing implementation using a specific strategy. In reality, while it is possible for an implementation strategy to result in full implementation of the intervention, it is likely that the implementation level takes some intermediate value. The actual value of implementation represents the additional incremental net benefit that will be provided to society after using an implementation strategy to improve the use of an intervention that is cost-effective. To calculate the value of actual implementation (equation (4)), the degree of uptake as a result of the implementation strategy (σ) replaces the assumption of 100% implementation in equation (3):

(4) n.(σ−p).NMB.

The next stage acknowledges the need to take account of opportunity cost by incorporating the resource use and cost for the implementation strategy and noting these will not be available for funding other health care services. Equation (5) shows how the incremental net benefit of implementation is calculated by subtracting the cost of the implementation strategy (*I*) from the actual value of implementation (equation (4)):

(5) n.(σ−p).NMB−I.

### Traditional Value of Implementation with Constant Marginal Costs and Benefits: Dynamic Form

The dynamic current value of implementation (VOImp [dynamic]) outlined by Walker et al.^28^ takes the following form:

(6) $$\sum_{t=1}^{T}\frac{n_{t}.p_{t}.\mathrm{NMB}}{{\left(1+r\right)}^{t-1}}.$$

The VOImp (dynamic) takes account of the value of implementation in different specified time periods (*t*) measured in annual increments. The time subscripts for the patient population (($n_{t}$) and proportion of patients currently receiving the intervention ($p_{t}$) allow for the size of the relevant patient population to change over time and for the provision of the intervention to naturally change by a process known as diffusion.^34^ The value gained from the intervention in future years also takes account of the future value of benefits using a discount rate (denoted by *r*). The value of perfect implementation (equation (7)), actual implementation (equation (8)), and the incremental net benefit of the intervention (equation (9)), representing the VOImp (dynamic), are then calculated by making the simple substitutions used in the static framework and adding the time subscript for σ:

(7) $$\sum_{t=1}^{T}\frac{n_{t}.\left(1-p_{t}\right).\mathrm{NMB}}{{\left(1+r\right)}^{t-1}}.$$

(8) $$\sum_{t=1}^{T}\frac{n_{t}.\left(\sigma_{t}-p_{t}\right).\mathrm{NMB}}{{\left(1+r\right)}^{t-1}}.$$

(9) $$\sum_{t=1}^{T}\frac{n_{t}.\left(\sigma_{t}-p_{t}\right).\mathrm{NMB}-I_{t}}{{\left(1+r\right)}^{t-1}}.$$

### Value of Implementation for Interventions with Varying Marginal Costs and Benefits: Static Form

Marginal costs and benefits are defined as those accrued from treating an additional patient. Incremental costs and benefits are defined as the difference in costs and benefits accrued from using the new intervention compared with a comparator (current practice). The marginal incremental costs and benefits are therefore defined as the additional costs and benefits experienced from treating 1 more patient with a new intervention rather than current practice. The assumption of constant marginal costs and benefits (see equations (4) and (9)) may provide misleading conclusions about the impact on relative cost-effectiveness of the new intervention if the marginal incremental costs and benefits vary at different levels of implementation. If the marginal incremental costs ($\Delta C_{p}$) and benefits ($\Delta H_{p}$) vary, this will result in a NMB that depends on the level of implementation:

(10) $$\mathrm{NM}B_{p}=k.\Delta H_{p}-\Delta C_{p}.$$

The current value of implementation in a static framework with varying costs and benefits (see equation (11)) is similar to that when using a static framework with a constant NMB (see equation (2)):

(11) $$n.p.\mathrm{NM}B_{p}.$$

Differences between the constant and variable NMB approaches begin to appear in the equation for calculating the perfect value of implementation (equation (12)). Since the NMB achieved at perfect implementation (σ=1) and current implementation (p) will be different, these cannot be collapsed in the way done to produce equation (3):

(12) $$n.(\mathrm{NM}B_{\sigma =1}-p.\mathrm{NM}B_{p}).$$

Similarly, separate NMB figures are required when calculating the value of actual implementation that takes change in marginal costs and benefits into account:

(13) $$n.(\sigma .\mathrm{NM}B_{\sigma }-p.\mathrm{NM}B_{p}).$$

The value of an implementation strategy is now dependent on the initial and final levels of implementation rather than simply being the constant net monetary benefit multiplied by the number of additional patients receiving the intervention. The VOImp (static) features a one-time implementation investment, and so a single cost of the strategy (*I*) can be subtracted to find the incremental net benefits of implementation:

(14) $$n.(\sigma .\mathrm{NM}B_{\sigma }-p.\mathrm{NM}B_{p})-I.$$

### Value of Implementation with Varying Marginal Costs and Benefits: Dynamic Form

Varying marginal costs and benefits can also be incorporated into the VOImp (dynamic) framework by allowing for the implementation specific levels of NMB (see equation (10)). The current value of implementation with varying marginal costs and benefits therefore becomes

(15) $$\sum_{t=1}^{T}\frac{n_{t}p_{t}\mathrm{NM}B_{p}}{{\left(1+r\right)}^{t-1}}.$$

It is necessary to take into account that implementation can naturally develop through the process of diffusion. The VOImp (dynamic) will be dependent on the implementation level and the expected relative cost-effectiveness, which represents the added net benefit of the proposed intervention compared with current practice. Therefore, the VOImp (dynamic) can potentially change over time without a specific strategy to change implementation. The value of perfect implementation (equation (16)) and value of actual implementation (equation (17)) can also be formulated by adding implementation-level specific NMB:

(16) $$\sum_{t=1}^{T}\frac{n_{t}(\mathrm{NM}B_{\sigma =1}-p_{t}\mathrm{NM}B_{p})}{{\left(1+r\right)}^{t-1}}.$$

(17) $$\sum_{t=1}^{T}\frac{n_{t}(\mathrm{NM}B_{\sigma }-p_{t}\mathrm{NM}B_{p})}{{\left(1+r\right)}^{t-1}}.$$

The time (representing a specific time period, for example, year) subscripts on the cost of the implementation strategy (*i*) and its level of implementation following an implementation strategy (σ) already allow for these factors to be nonlinear over time, and this may provide an additional source of nonlinearity in relative cost-effectiveness representing the added value of the intervention in different time periods:

(18) $$\sum_{t=1}^{T}\frac{n_{t}(\sigma_{t}\mathrm{NM}B_{\sigma }-p_{t}\mathrm{NM}B_{p})-i_{t}}{{\left(1+r\right)}^{t-1}}.$$

### Case Study: 70-Gene Recurrence Score Test for Breast Cancer

This section describes a case study to show the impact of considering the value of implementation in precision medicine while taking account of varying marginal costs and benefits. This example builds on the work of Retèl et al.^35^ that used a published decision-analytic (Markov) model-based CEA of the 70-gene recurrence score test (MammaPrint) to guide treatment selection of early chemotherapy for women at risk of breast cancer recurrence. MammaPrint is a test that provides a score indicating the risk of breast cancer recurrence. This score can be used to inform treatment options that can be stratified based on this score. Women at high risk of recurrence can receive adjuvant chemotherapy while those at a low estimated risk can be monitored without experiencing the potential side effects of chemotherapy.

Retèl et al.^35^ aimed to anticipate the barriers and facilitators to introduce MammaPrint into clinical practice and developed a range of scenarios that might occur during implementation. Retèl et al.^35^ used the Delphi method to bring together stakeholders in the potential implementation of the MammaPrint test to understand the degree of consensus about the potential barriers and facilitators to its introduction and to determine how likely it would be that each barrier or facilitator would occur. An initial list of 10 barriers and facilitators was produced, and these were then condensed into 3 critical barriers that were included in the Markov model: technical failure, noncompliance with discordant test results (results that do not align with the clinician’s perceptions of the patient’s risk), and uptake by clinicians. The most significant barrier proved to be the uptake of the intervention (see Table 1), which produced significantly different incremental cost-effectiveness ratios at different levels of use of test results by clinicians in decision making. MammaPrint was observed to not meet accepted thresholds of relative cost-effectiveness at the lowest level of implementation (3%) but became more cost-effective with increasing levels of implementation due to rising marginal incremental benefits and falling marginal incremental costs.

**Table 1:** Incremental Cost-Effectiveness Ratios of MammaPrint at Different Implementation Levels^a^

Assumed Proportion of Use of Test Results by Clinicians	Marginal Incremental Benefits (QALYs)	Marginal Incremental Cost (€)	Marginal ICER (Compared with Current Practice)
3%	0.001	1940	€1.9 million per QALY gained
50%	0.0728	1630	€22,388 per QALY gained
92%	0.1492	1171	€7,853 per QALY gained

ICER, incremental cost-effectiveness ratio; QALY, quality-adjusted life year.

a Source: Retèl et al.^35^

The observed varying marginal incremental costs and benefits of MammaPrint arose due to the presence and gradual removal of the barriers to implementation. For example, in the event of technical failure, it was assumed that 10% of the cost of the MammaPrint was spent with no concurrent benefit gained. Similarly, when test results were returned but the clinician did not use the result to change practice, a cost was incurred but no benefit gained. These factors result in a higher initial cost per patient of the test when MammaPrint was implemented due to technical failure and a lower benefit per patient as not all of the tests were used to change clinical practice. As implementation improves over time and these barriers are removed, the marginal incremental costs reduce and the marginal incremental benefits increase, thereby improving the relative cost-effectiveness of MammaPrint.

#### Static VOImp analysis of MammaPrint with variable marginal costs and benefits

The selected case study used the results of a published state transition Markov model-based cost-effectiveness analysis in which the estimated variation in the marginal costs and benefits was driven by including specific barriers and facilitators to the introduction of MammaPrint into clinical practice.^36^ The process, driven by the structure and assumptions of the decision-analytic model, of how these barriers and facilitators generate varying marginal costs and benefits is likely to be complex. Ideally, access to the decision-analytic model is required to predict the marginal costs and benefits of precision medicine at different levels of implementation. A working version of the state transition Markov model produced by Retèl et al.^35^ was not available in the public domain. Therefore, to provide an illustrative example for this study in the absence of a decision-analytic model, the outputs of the analysis produced by Retèl et al.^35^ were used. Using these outputs, a simple meta-model was created using ordinary least squares regressions to predict the marginal costs and benefits of the intervention at different levels of implementation of MammaPrint. The 3 data points for the marginal incremental costs and benefits shown in Table 1 (taken from Retèl et al.^35^) were used to estimate a linear function (ordinary least squares [OLS] regression) that approximates the benefits and costs as a function of the implementation level (*p*) (equations (19) and (20)). Microsoft Excel (Microsoft, Redmond, WA) was used to perform the OLS regression.

(19) $$\Delta H_{p}=0.1662p-0.006.$$

(20) $$\Delta C_{p}=1996-860p.$$

Equation (19) had an *R*^2^ value of 0.998, and equation (20) had an *R*^2^ value of 0.980. These 2 *R*^2^ values indicate the estimated OLS regression was a good approximation of the relationship between the implementation level and the marginal incremental costs and benefits generated by the underlying state transition Markov model. These equations suggest that as implementation of MammaPrint increases, the marginal health gains will rise and the marginal costs will fall. More generally, a decision-analytic model could be run to generate the estimates of marginal health gains and costs. Using equation (20), in this example, the variable NMB can then be predicted as a function of the implementation level (*p*) of MammaPrint by substituting equations (19) and (20) and a threshold value (*k*) into equation (10). Using this approach, with a threshold value of €20,000 per QALY, results in a formula for the variable NMB:

(21) $$\mathrm{NM}B_{p}=4184p-2116.$$

The positive coefficient before the implementation variable *p* suggests that there are increasing returns to scale associated with MammaPrint. In other words, as the extent of implementation of MammaPrint in the population rises, the net monetary benefit of an additional patient receiving the test-treat strategy also increases. This static VOImp analysis has made the simplifying assumption that changes in marginal costs and benefits would be driven by the implementation level affecting cost by economies of scale and consequences by learning effects. The analysis also assumed that each patient would receive the same incremental benefit from MammaPrint at the same incremental cost. In practice, the incremental marginal costs and benefits of MammaPrint will depend on a range of factors other than the degree of uptake into the population.

The point at which MammaPrint becomes cost-effective, in this scenario, can be calculated by finding the cutoff point of *p* when NMB is set equal to zero. Estimating this cutoff point suggests that an implementation level of over 51% is required for MammaPrint to constitute a cost-effective use of resources at a threshold of €20,000 per QALY gained.

The marginal costs and benefits at the highest implementation level presented in Retèl et al.^35^ (92%) were used to calculate the NMB when marginal costs and benefits were assumed to be constant to be used as the comparator option. This approach was taken because traditional cost-effectiveness analysis provides estimates that represent the long-run cost-effectiveness of the precision medicine.^14^ These traditional estimates of cost-effectiveness assume that all patients will receive the precision medicine, and factors such as short-run fixed costs are not considered.^37^ The costs attributed to the intervention at the period furthest forward in time from start of the decision analysis therefore best represent the (long-run) results that would be produced by a traditional CEA as they will be as close to the optimal use of the intervention as possible.

#### Static value of a strategy to improve implementation of MammaPrint with variable marginal costs and benefits

The VOImp (static) of any implementation strategy will be dependent on the initial and final level of implementation in addition to the magnitude of the change in implementation over time. Using equation (4), assuming a constant NMB and a 20% point rise in the degree of implementation of MammaPrint results in a constant value of €3,626,000 per QALY gained in a population of 10,000 women (Table 2). In practice, the nonlinear nature of the marginal costs and benefits, because of the structure and assumptions within a decision-analytic model, of MammaPrint will result in a change in the relative cost-effectiveness that is dependent on the initial baseline and final level of implementation. Raising implementation from the baseline value of 20% to 40% had a low level of relative cost-effectiveness, implying that the resources direct to the implementation strategy may have been used to better effect in a different area of the health system. In contrast, moving from a baseline level of 40% to 60% had a substantially bigger impact on relative cost-effectiveness, which in absolute terms meant MammaPrint moving from being a cost-ineffective to a cost-effective use of resources at a threshold of €20,000 per QALY gained.

**Table 2:** VOImp (Static) Accounting for the Baseline and Final Level of Implementation of MammaPrint in a Population of 10,000 Women

		Constant Marginal Costs and Benefits (*N* = 10,000)		Varying Marginal Costs and Benefits (*N* = 10,000)	
Description	Formulae	Baseline Implementation: 20%^a^ Final Implementation: 40%	Baseline Implementation: 40% Final Implementation: 60%	Baseline Implementation: 20%^a^ Final Implementation: 40%	Baseline Implementation: 40% Final Implementation: 60%
Current value of implementation before introducing an implementation strategy	$n.p.\mathrm{NM}B_{p}$	€3,626,000	€7,252,000	€−2,558,400	€−1,769,600
Current value of implementation after introducing an implementation strategy	$n.\sigma .\mathrm{NM}B_{\sigma }$	€7,252,000	€10,878,000	€−1,769,600	€2,366,400
Actual value of implementation	$n(\sigma .\mathrm{NM}B_{\sigma }-p.\mathrm{NM}B_{p})$	€3,626,000	€3,626,000	€788,800	€4,136,000

a The baseline level of 20% was assumed using the estimated incidence of breast cancer in the Netherlands of approximately 14,000 cases per year, of which approximately 80% of women could benefit from MammaPrint.^53,54^ We therefore assumed approximately 10,000 women a year could benefit from the test.

#### Dynamic VOImp analysis of MammaPrint with variable marginal costs and benefits

**Figure 1:**
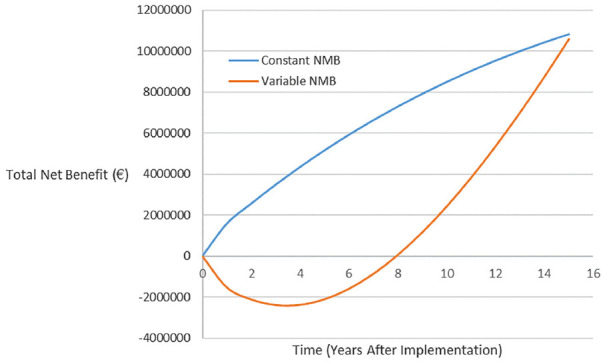
Current value of implementation (dynamic) for MammaPrint assuming constant or variable net monetary benefit (NMB).

The VOImp (dynamic) of MammaPrint is now shown. It is necessary to make some key assumptions. Specifically, it was necessary to assume that the population of eligible patients does not change over time and there is a diffusion rate of 6 percentage points per year in that population. This assumption reflected the time frame for implementation outlined in Retèl et al.^35^ In this example, a discount rate of 3% per year was applied to the net health benefit. Figure 1, with the actual values shown in Table 3, illustrates how the current value of implementation of MammaPrint differed substantially depending on whether constant or varying NMB was assumed.

**Table 3:** Current Value of Implementation (Dynamic) for MammaPrint Assuming Constant or Variable Net Monetary Benefit

Year	Constant Net Monetary Benefit^a^	Varying Net Monetary Benefit^a^
1	€1,584,175	−€1,519,899
2	€2,563,390	−€2,104,440
3	€3,484,219	−€2,377,955
4	€4,349,233	−€2,366,098
5	€5,160,902	−€2,093,055
6	€5,921,600	−€1,581,619
7	€6,633,607	−€853,258
8	€7,299,115	€71,824
9	€7,920,227	€1,174,623
10	€8,498,966	€2,437,277
11	€9,037,273	€3,843,010
12	€9,537,013	€5,376,077
13	€9,999,975	€7,021,714
14	€10,427,877	€8,766,089
15	€10,822,370	€10,596,252
**Total**	**€103,239,942**	**€26,390,541**

a Assuming a population size of 10,000 patients.

Under an assumption of constant NMB, MammaPrint gradually diffuses into practice and provides additional value for the health system at a constantly increasing rate, albeit with diminishing returns due to impact of the discount rate that considers the effect of time preference. Taking account of the potential for variable NMB, it can be observed that, in the initial years following implementation, MammaPrint was not a cost-effective use of resources. Over the first 7 years, the total losses of MammaPrint accrue to a total monetary loss of €12,896,324. The resources allocated to MammaPrint over the first 7 years could have been used to gain approximately 645 QALYs by funding other interventions in the health system. MammaPrint does become cost-effective at 9 years and produces a marginally increasing net benefit in each year, but the total value accrued over these 9 years is a fraction (26%) of that predicted when using a constant NMB.

#### Dynamic value of a strategy to improve implementation of MammaPrint with variable marginal costs and benefits

**Figure 2:**
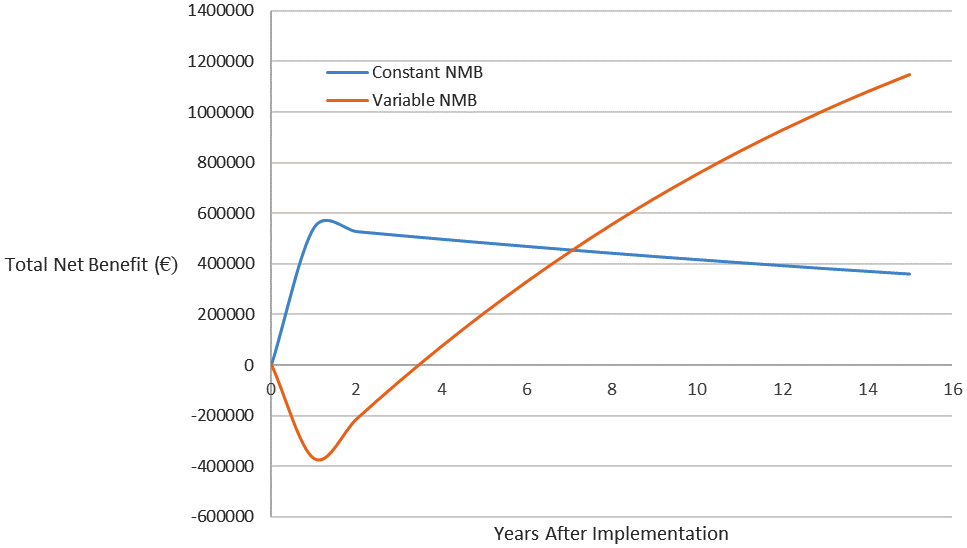
Actual value of an implementation strategy that improves uptake by 3 percentage points. NMB, net monetary benefit.

The dynamic VOImp for MammaPrint can be calculated for a hypothetical implementation strategy that raises implementation of the test-treat strategy by 3 percentage points in the first year (from 9% to 12%), and it is assumed this effect is sustained for the following 14 years. A discount rate of 3% per year was applied to the net health benefit. The value, in terms of the total incremental net benefit resulting from the implementation strategy, is shown graphically in Figure 2 and numerically in Table 4.

**Table 4:** Actual Value of Implementation (Dynamic) for MammaPrint Assuming Constant or Variable Net Monetary Benefit That Improves Uptake by 3 Percentage Points

Year	Implementation Level without Implementation Strategy	Implementation Level with Implementation Strategy	Constant Net Monetary Benefit^a^	Varying Net Monetary Benefit^a^
1	0.09	0.12	€543,900	−€371,208
2	0.15	0.18	€528,058	−€214,159
3	0.21	0.24	€512,678	−€65,944
4	0.27	0.30	€497,746	€73,819
5	0.33	0.36	€483,248	€205,496
6	0.39	0.42	€469,173	€329,441
7	0.45	0.48	€455,508	€445,991
8	0.51	0.54	€442,240	€555,472
9	0.57	0.60	€429,360	€658,197
10	0.63	0.66	€416,854	€754,467
11	0.69	0.72	€404,713	€844,570
12	0.75	0.78	€392,925	€928,785
13	0.81	0.84	€381,481	€1,007,378
14	0.87	0.90	€370,369	€1,080,604
15	0.93	0.96	€359,582	€1,148,711
**Total**			**€6,687,834**	**€7,381,619**

a Assuming a population size of 10,000 patients.

The value of this implementation strategy for MammaPrint, which increases uptake by 3 percentage points in each time period, is broadly constant in each year when NMB is assumed to be constant. This impact is observed because the same amount of additional health and cost is gained from treating an additional 3% of patients in each year regardless of the number of patients already receiving the MammaPrint intervention. Under a variable NMB, the value of such an implementation strategy is negative in early periods but will increase over time. This finding was because at low levels of implementation, the intervention is not cost-effective, and so the additional patients receiving the intervention will mean increases in the societal loss arising from implementation. Over time, the additional implementation eventually decreases the marginal costs and increases the marginal benefits for patients, meaning that there are increasing returns in later time periods. The overall result is that the implementation strategy is worth nearly €700,000 more when accounting for variable NMB despite the fact that it results in a societal loss soon after its launch.

## Discussion

This study has illustrated a general approach to show how existing value of implementation methods can be adapted to account for varying marginal incremental costs and benefits in a specific case study focusing on precision medicine. The study has adapted existing value of implementation methods, using the outputs from a decision-analytic model-based analysis, to understand the potential impact on the cost-effectiveness of strategies to improve the implementation of precision medicine. This approach differs from traditional approaches that present the results from a decision-analytic model as incremental cost-effectiveness ratios on a cost-effectiveness plane showing the threshold for cost-effectiveness,^38^ by taking account of the total number of patients taking up precision medicine as a proportion of the number of patients eligible for treatment.^38^ Taking account of the level of implementation allows the total societal net benefit of precision medicine to be determined. Calculating the incremental cost-effectiveness ratio (ICER) only allows a yes or no decision to be made regarding acceptance of precision medicine dependent on a stated threshold.^33^ Precision medicine can appear to be cost-effective but not maximize total societal net benefit if it is only implemented in a proportion of the total eligible patient population.

In this study, we have built on a published decision-analytic model-based cost-effectiveness analysis of MammaPrint and shown that assuming constant marginal net costs and benefits may produce misleading results when assessing the relative cost-effectiveness of implementation strategies. The need to take account of the impact of implementation on relative cost-effectiveness is important to any new health care intervention. However, the exemplar of precision medicine has specific and known multiple barriers to timely and effective implementation.^15,17^ Varying marginal costs and benefits may be a particularly prevalent issue when introducing exemplars of precision medicine. Such interventions often involve some kind of test or stratifying mechanism to identify which patients may benefit or be harmed by a treatment or to predict disease course. Previous studies have identified a lack of testing capacity as a barrier to precision medicine, and other issues, such as the need to develop logistical mechanisms to deliver tests in a timely manner, may also impede implementation.^15,16,39,40^ As such, it is possible that the significant upfront investments in capacity would mean that it would not be cost-effective to use the precision medicine to treat a small eligible patient population. Another barrier may be a lack of training or guidelines in the use of precision medicine, and as such, there may also be learning effects where the outcomes of testing and treatment improve as test providers and clinicians gain experience in providing the interventions.^17,39^

The relative cost-effectiveness and net marginal value of precision medicine will depend on the level of implementation. Without using implementation strategies, it is possible that losses will accrue over multiple time periods before implementation reaches a cost-effective level. Information about the required level of implementation would not be provided by a conventional CEA, which would have judged the precision medicine to be universally cost-effective based on the average ICER calculated for treating all of the patients in the long run.

It is necessary, but not sufficient, to use estimates of the long-run cost-effectiveness to understand whether an intervention is an efficient use of health care resources. In some instances, the decision problem is more appropriately expanded to consider the implementation of interventions that appear to be cost-effective. This is likely to be of particular relevance in precision medicine, which involves the implementation of 2 elements, a “test” and a “treatment,” into existing pathways of care. In such instances, estimates of cost-effectiveness should allow for varying marginal costs and benefits and differential cost-effectiveness at different levels of implementation of precision medicine. These approaches could use static models with costs and benefits that are described as a function of implementation levels and producing estimates of cost-effectiveness at different levels or dynamic, multicohort models to capture the impact of changing marginal costs and benefits over time.^18^

In applied studies using the methods outlined in this article, the incremental costs and consequences of a specific example of precision medicine will be estimated using a decision-analytic model. Using the framework of a decision-analytic model will allow the analyst to vary the marginal costs and benefits depending on prespecified factors such as capacity constraints, economies of scale, learning effects, and patient characteristics. The computational effort, in terms of technical skills and computer processing power, in adapting existing decision-analytic model-based cost-effectiveness analysis is minimal. The approach involves making modest adaptations to the structure of existing decision-analytic models and presenting the outputs in a particular way. However, this approach does require an understanding of potential barriers and facilitators to implementation, which may require mixed-methods approaches such as the Delphi method or qualitative methods such as semistructured interviews.^41,42^

The method proposed in this article will be relevant to a number of applications and specified decision problems. Clear criteria will be needed to define the scope of the relevant decision problem that need to take account of the value of implementation. Such criteria will be needed to identify potential interventions that are likely to exhibit varying marginal incremental costs and consequences. For example, vaccines exhibit positive externalities that arise because the vaccine protects not only the individual against infection but also other people within a population because the spread of disease can be halted. The magnitude of the additional benefits accrued will reduce as more people are vaccinated and therefore do not benefit from the externality. Another potentially relevant example is in the implementation of a new surgical technique. As surgeons gain experience in the surgical technique, the length of time required to perform the operation, and hence costs, may reduce in addition to improvements in patient outcome. These 2 examples illustrate some potential situations in which marginal incremental costs and benefits may arise, indicating that the method proposed in this study may be valuable.

Other causes of variable marginal costs and benefits in an intervention include the following: economies or diseconomies of scale may affect the marginal cost per patient as implementation increases,^43^ economies of scope may arise as resources can be shared over multiple interventions,^43^ learning curves may mean that clinicians’ experience and skill in using an intervention increase as they treat more patients and therefore patient health outcomes increase,^44^ and prioritization of sicker patients may lead to decreasing marginal health benefits.^45^

The issue of variable marginal costs and benefits has previously been explored by Lord et al.^45^ with regards to the implications of nonlinear cost-effectiveness frontiers for funding decisions. For a convex frontier (e.g., for an intervention where patients are prioritized by likely benefit, leading to decreasing returns to scale), the optimal decision may actually be to fund a portion of the new intervention while keeping some patients on the old intervention. These authors found that when phenomena such as decreasing marginal costs caused the frontier to be concave, the decision to either fund the intervention for all patients or to fund it for none would always be the optimum in terms of net benefit. They concluded that “partial implementation will not be cost-eﬀective, and hence estimation of the expansion path (the movement from the old to the new intervention) will not be productive.”^45^ However, it is possible that an imperfect implementation of an intervention that is cost-effective at the population level will be cost-effective when compared with only funding the existing intervention. Similarly, some levels of implementation of the new intervention will not be cost-effective. Whether the implementation level of the new intervention is cost-effective or not will depend on the point at which the cost-effectiveness frontier crosses the threshold of relative cost-effectiveness (e.g., £20,000 per QALY gained) for the health system. Therefore, while partial implementation will never be the most cost-effective combination of the 2 interventions in concave frontiers, in reality, the level of implementation is critical to the cost-effectiveness of the intervention.

One previous study by Grimm et al.^46^ allowed for varying marginal costs in assessing the cost-effectiveness of implementation of a preterm birth screening test but not in a value of implementation framework. Grimm et al.^46^ modeled the impact of falling future prices on the cost-effectiveness of the screening test. In their case study, the cost of the screening test fell to 90% of the previous value every time the number of patients receiving it doubled. The ICER reduced it by up to 46% with increasing implementation. The authors suggested that to account for this varying marginal cost in decision making, a dynamic ICER could be produced reflecting the average ICER across all time periods but did not go on to provide this analysis.

### Limitations

This study has used an example of precision medicine where changes in the estimated marginal costs and benefits were modeled, in the absence of access for a fully executable decision-analytic model, as approximately linear functions of changes of implementation level. While this simplified the mathematical solution to the problem and is useful in demonstrating the consequences of varying marginal costs and benefits, in reality, marginal costs and benefits may not be a smooth function of implementation. For example, a number of capacity investments may be required to increase patient access to elements (test and/or treatment) of precision medicine. Each capacity investment has its own cost and effect in terms of increasing implementation. Over the course of achieving full implementation, a current value of implementation curve (e.g., Figure 1) may, in practice, comprise a number of different, linked curves with different slopes. While there may be an apparent overall effect of increasing or decreasing net monetary benefit, a formula to predict such changes by modeling NMB as a function of implementation may not represent a good approximation. In such cases, the marginal costs and benefits at each implementation level of interest may have to be estimated directly based on input data or a more complex underlying model such as a discrete event simulation.

A key assumption made in the example presented in this article is that the required parameters were known with absolute certainty. In addition, the case study took the perspective of determining the value of implementing an intervention after it has been approved as cost-effective based on estimates of the cost-effectiveness of MammaPrint at full implementation. The aligns with current approaches to HTA that implicitly take a “long-run” perspective while failing to account for differential cost-effectiveness in the short term.^14,47^ Given that the decision to adopt has been taken based on certain evidence, the counterfactual used in this example is continued use of the comparator that produces no incremental net benefit.

In practice, accounting for uncertainty is critical when determining the value of implementing interventions and whether it is necessary to obtain more information about a specific parameter through further research. It is difficult to quantify the value of an implementation strategy when there is a probability that the intervention itself is not cost-effective. The presence of varying marginal costs and benefits will have significant and complex consequences for such applications of VOImp and VOI methods by introducing an additional layer of uncertainty. In addition to estimating the distribution of costs and benefits for the intervention and comparator, the analyst will also have to forecast how these costs and benefits will change depending on implementation levels. In the example presented in this article, the levels of implementation for which the intervention would not be cost-effective due to high marginal costs and low marginal benefits would also be associated with a degree of uncertainty. Implementation strategies will only be shown to be of added value if there is a sufficiently large probability that the strategy will result in a distribution of implementation levels at which introducing the precision medicine becomes cost-effective at a predefined threshold.

In the presence of uncertainty, value of information methods can be used alongside VOImp to determine whether it is better to invest in implementation or better evidence as to the values of the parameters in the model.^29,31,48^ Such approaches determine the optimal combination of research and implementation of an intervention compared to the counterfactual of implementing the comparator with the highest net benefit given current levels of information.^30^

The presence of varying marginal costs and benefits will also have an impact on the expected value of further research for precision medicine. For example, there may be significant value in identifying the levels of implementation at which precision medicine would not be cost-effective by providing better estimates of the marginal costs and benefits. This would minimize the risk of a resulting loss to society by introducing a strategy to deliver precision medicine that was not cost-effective or not cost-effective at the observed degree of uptake in clinical practice.

This study used an example of precision medicine with marginally decreasing costs and increasing benefits, with the overall effect of producing increasing net monetary benefit with increasing implementation. However, it is possible that different patterns of nonlinearity are exhibited in marginal costs and benefits. For example, a strategy to deliver precision medicine with marginally increasing costs and/or decreasing benefits will have an opposite pattern of total net benefit with implementation. At lower levels of implementation, precision medicine will be more cost-effective, potentially becoming cost-ineffective at higher levels due to the decreasing returns to net monetary benefit. For example, prescription of oral preexposure prophylaxis (PrEP) in individuals at risk of infection with human immunodeficiency virus provides a benefit to the patient in that the risk of infection is dramatically reduced but may also provide a positive externality in preventing that individual from passing on the virus to others.^49^ However, the use of PrEP may be more beneficial in those patients whose behaviors put them at greater risk of infection, and as such, the cost-effectiveness of the intervention may decrease with the reducing risk status of the patients treated. As such, a strategy to stratify the population by risk of infection may be useful to ensure the intervention is offered in a cost-effective manner.

For some examples of precision medicine, with decreasing returns to scale, there may be concerns about the equity of only providing the intervention to a smaller number of individuals in order for provision of precision medicine to remain cost-effective. While the equity-efficiency tradeoff is an extensive existing research area,^50–52^ the value of implementation approach outlined in this article may help to quantify the total loss in net benefit that would be incurred from increasing implementation from the maximum cost-effective level to full implementation.

It is also conceivable that an intervention could exhibit both increasing and decreasing returns to net monetary benefit at different levels of implementation of precision medicine. For example, a strategy to deliver precision medicine involving mutation testing to guide a cancer treatment may have high initial marginal costs due to the need to invest in testing equipment and training. As these costs are divided across increasing numbers of patients, their impact decreases, making the intervention more cost-effective and creating a positive total net benefit. However, higher marginal costs and slower turnaround times may be faced in sending samples from rural hospitals to laboratories in cities. This may lead to decreasing marginal returns when treating these patients and achieving full implementation. In such circumstances, it is possible that there is both a minimum and maximum level of efficient implementation for the intervention.

It is also important to note that the pattern of nonlinearity in the marginal costs and benefits of any existing interventions that are to be disinvested from to fund precision medicine will also have implications for the efficiency of the health service. If an existing intervention to be defunded exhibits increasing returns to scale, then the initial disinvestment will cause a significant loss in health for minimal cost savings. An intervention with decreasing returns could be defunded with larger savings gains for smaller health losses.

## Concluding Remarks

This study used a specific case study focusing on precision medicine to show the importance of going beyond an evaluation of the long run cost-effectiveness of an intervention and the limiting assumption of a constant net monetary benefit. Anticipating the pattern of nonlinearity in marginal costs and benefits and its impact on the value of implementation of precision medicine is important to ensure that otherwise cost-effective interventions are not implemented in a way that causes a net health loss to society. The use of value of implementation analysis in the context of precision medicine to evaluate implementation-improving strategies is also likely to produce significantly biased results if varying marginal costs and marginal benefits are not incorporated.
